# Causality Implications for Absorption by EM Metasurfaces

**DOI:** 10.3390/nano15110793

**Published:** 2025-05-25

**Authors:** Constantinos Valagiannopoulos

**Affiliations:** School of Electrical & Computer Engineering, National Technical University of Athens, GR-15780 Athens, Greece; valagiannopoulos@ece.ntua.gr

**Keywords:** absorption, causality, Lorentz oscillator, metasurface

## Abstract

A causal electromagnetic (EM) metasurface is backed by a lossless substrate and partially absorbs obliquely incoming rays. The integral of the absorbed power along the entire frequency axis is analytically evaluated, and the obtained sum rules indicate the global absorption by such a generic configuration. The beneficial influence of the plasma frequency and damping factor on the total absorbance score as well as the opposite effect of the angle of excitation, is noted. An overall lossless behavior at the incidence direction where the propagating waves into the substrate turn into evanescent is identified, once the magnetic field is parallel to the interface. The reported results can be useful in the tailoring of spectrally dependent absorption by a whole class of planar structures and, accordingly, in the forward and inverse design of lossy photonic metasurface setups.

## 1. Introductory Comment

Sum rules, namely, formulas of static quantities expressed as integrals over a continuous variable characterizing the excitation of a dynamical system, can unveil critical information regarding its behavior; it can happen within many scientific branches, from quantum mechanics and fluid dynamics to optoelectronics and electromagnetics. Indicatively, the consistency relations imposed by the long range of the Coulomb force end up as sum rules for the correlation functions of charged fluids [1], and similar Gaussian formulas for finite energy are derived in the case of quantum chromodynamical setups [2]. Moreover, explicit calculations involving the energy–momentum tensor in quantum electrodynamics [3], the waveguide electroabsorption in quantum well structures [4], and the maximum possible off-resonant susceptibilities of nonlinearly coupled molecules [5,6], have been performed. Importantly, similar formulas provide the characteristic ground state property in any semiconductor or insulator [7] to identify the spectral limits for disordered condensed matter interfaces [8] and to describe the exciton binding energy in perovskite solar cells [9].

In electrodynamics, a crucial constraint that leads to the sum rules defining the limits on the performance of the respective systems is the principle of causality. Indeed, the demand for the input to precede the output has led to certain bounds in the radiation of antennas (Chu limit [10]) and in the matching of impedances (Bode–Fano limit [11]). Even the fundamental Kramers–Kronig relations express the need for causality and can be updated to dictate the development of waves into double-negative media [12] and be connected with passivity (excluding setups based on gain media [13]) within the paradigm of metamaterials [14]. Similarly, it has been found that maximum cloaking efficiency cannot be achieved at arbitrarily extended bands [15] and that the Drude model has issues when applied to time-modulated media [16]. Finally, the principle of causality can be extremely helpful in inverse problems when determining the constituent spectral parameters of unknown samples with uncertainties [17], matched anisotropic absorbers [18], and highly dispersive materials [19].

The aim of this work is to apply causality constraints on EM absorption, which is a particularly important operation behind numerous applications such as wide-angle stealth operation [20], bianisotropic tunable isolation [21,22], and wideband RCS reduction [23]. In particular, we consider a metasurface whose homogenized surface conductivity obeys the causal Lorentz model; it is grown on a lossless substrate while being excited from the other side, by a beam traveling into free space. The sum rule for global absorption at a specific angle of illumination is defined as the integral of monochromatic absorption along the entire frequency axis. Closed-form expressions are derived for both polarizations as long as the waves into the substrate are propagating; in case they are evanescent, approaches based on efficient numerical integrations are proposed. The metric is maximized for lossless plasmonic substrates, and, once the magnetic field is parallel to the interface, it vanishes around the critical angle where propagating waves are converted into evanescent ones. The reported sum rules provide causality bounds on the absorption by Lorentzian metasurfaces and can be critical when evaluating the respective matching scores for similar classes of devices across specific bands.

## 2. Mathematical Formulation

### 2.1. Causal Metasurface

**Figure 1:**
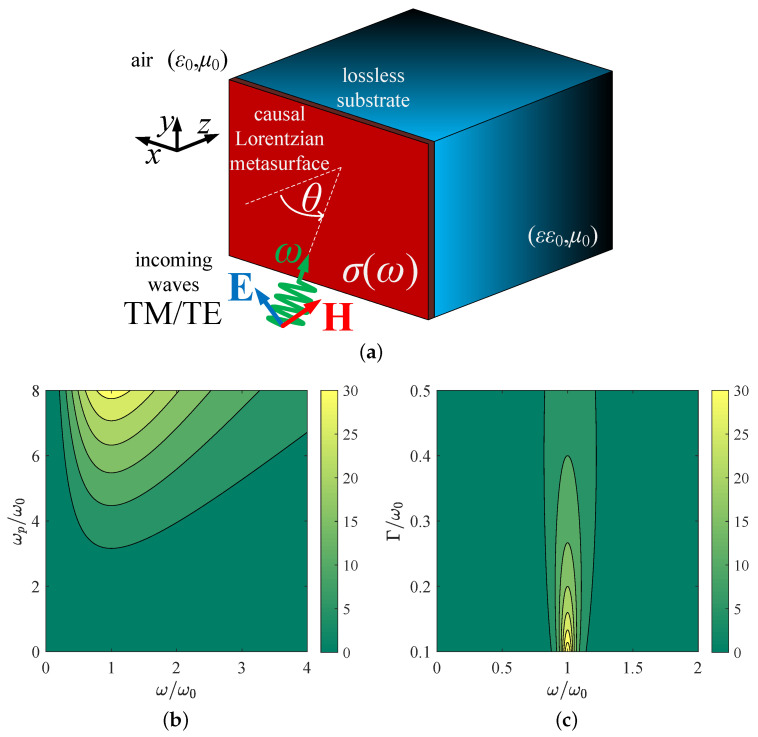
(**a**) A metasurface of causal surface conductivity σ(ω) grown on the top of a lossless dispersionless substrate of relative permittivity ε is excited by an obliquely incident plane wave traveling along a direction forming an angle θ with the normal-to-interface axis *z*. (**b**) The real part of the normalized surface conductivity $\sigma \eta_{0}$ with respect to the operational frequency $\omega /\omega_{0}$ and the plasma frequency $\omega_{p}/\omega_{0}$ of the Lorentzian model ($\Gamma =2\omega_{0}$). (**c**) The real part of the surface conductivity $\sigma \eta_{0}$ with respect to operational frequency $\omega /\omega_{0}$ and the losses frequency $\Gamma /\omega_{0}$ of the Lorentzian model ($\omega_{p}=2\omega_{0}$).

We consider the setup depicted in Figure 1a, where a metasurface is grown on a lossless base with relative permittivity ε∈R and is excited by an obliquely incident plane wave whose direction of propagation forms an angle θ with the normal-to-interface *z* axis. The wave travels into vacuum $\left(\varepsilon_{0},\mu_{0}\right)$ with time dependence exp(+iωt), while the surface conductivity of the metasurface obeys a causal Lorentz model for dispersion, namely,

(1) $$\begin{array}{c} \sigma \left(\omega \right)=\frac{1}{i\eta_{0}}\frac{\left(\omega /\omega_{0}\right){\left(\omega_{p}/\omega_{0}\right)}^{2}}{{\left(\omega /\omega_{0}\right)}^{2}-1-i\left(\omega /\omega_{0}\right)\left(\Gamma /\omega_{0}\right)}. \end{array}$$

The frequency $\omega_{p}$ is the plasma frequency, being proportional to the number of free charge carriers across the metasurface, the quantity $\omega_{0}$ is the natural frequency of the oscillator model representing each atom, and the frequency Γ indicates the losses originating from the collision of electrons with the crystalline structure. Note that the surface conductivity is a function of only two parameters ($\omega_{p}/\omega_{0}$, $\Gamma /\omega_{0}$); the normalized frequency $\omega /\omega_{0}$ while $\eta_{0}=\sqrt{\mu_{0}/\varepsilon_{0}}=120\pi \;\Omega$ is the constant wave impedance into free space. The real part of surface conductivity is always positive, a feature that marks the absorptive nature of the metasurface. The imaginary part of σ, determines the reactive behavior of the setup; in particular, if Im[σ]>0, it exhibits a dielectric (capacitive) response, while if Im[σ]<0, the device interacts in a plasmonic (inductive) manner [24]. Given the fact that the complex surface impedance $Z_{s}\left(\omega \right)$ is prevalent in practical modeling and experimental interpretations, it is meaningful to point out that $Z_{s}\left(\omega \right)=1/\sigma \left(\omega \right)$, and the respective definitions can be made in terms of its own real and imaginary parts.

The causal model for the surface conductivity adopted by (1) is quite common when dealing with effective boundary conditions. In particular, metasurfaces designed by the arrangement of electrically small resonant nanoparticles, cylindrical or with edges [25], can show such Lorentz-type dispersion [26]. Similar causal responses are utilized when examining the interplay of multipolar resonances or investigating bound and quasi-bound states towards enhanced light–matter interactions [27]. Importantly, Lorentzians are routinely used to describe the frequency-dependent behavior of equivalent circuits, which help in designing broadband nanometer-scale absorbers operated in the optical spectrum [28].

In Figure 1b, we present the unitless quantity Re[ση0] across a map with the normalized operational frequency $\omega /\omega_{0}$ on the horizontal axis and the normalized plasma frequency $\omega_{p}/\omega_{0}$ on the vertical axis. One directly observes that the real part of surface conductivity increases with $\omega_{p}/\omega_{0}$, which is natural since more charge carriers become available to absorb the incoming power via particle collisions as they move tangentially to the interface z=0. In addition, a higher boost is noted for $\omega ≅\omega_{0}$, where the incoming wave oscillation is synchronized with the natural resonance of the Lorentzian model. In Figure 1c, again, the quantity Re[ση0], which is responsible for the absorption of the metasurface, is shown across a plane defined by half the band of Figure 1b, and the dimensionless losses $0.1<\Gamma /\omega_{0}<0.5$. Surprisingly, the lower the losses, the smaller the Re[ση0] for the considered range; furthermore, the most substantial values for the represented quantity are again recorded when $\omega ≅\omega_{0}$. Indeed, if one separates the real part from (1) and takes the derivative of Re[ση0] with respect to $\omega /\omega_{0}$, one finds that the function decreases for $\sqrt{1+{\left[\Gamma /\left(2\omega_{0}\right)\right]}^{2}}-\Gamma /\left(2\omega_{0}\right)<\omega /\omega_{0}<\sqrt{1+{\left[\Gamma /\left(2\omega_{0}\right)\right]}^{2}}+\Gamma /\left(2\omega_{0}\right)$.

It is, therefore, shown that, if the causal metasurface is operated close to the natural frequency of the oscillators $\omega =\omega_{0}$, the lossy part of σ is negatively influenced by an increase in Γ. In particular, the larger the Γ gets selected, the larger the bandwidth $\Gamma /\omega_{0}$ of this effect becomes, around a central oscillating frequency $\omega =\omega_{0}\sqrt{1+{\left[\Gamma /\left(2\omega_{0}\right)\right]}^{2}}.$ The natural trend that Γ automatically enhances Re[σ] is valid only when we operate the system away from the natural frequency of oscillations $\omega_{0}$. In that spectral vicinity, the denominator of σ contains only a term of Γ, which suppresses the magnitudes of both parts of the surface conductivity. In other words, for $\omega =\omega_{0}$, we obtain Re[ση0]∼ω0/Γ (due to the vanishing $\left[{\left(\omega /\omega_{0}\right)}^{2}-1\right]$ term in the denominator of (1)) and, accordingly, lower losses do not correspond to smaller Re[σ]. It should be also pointed out that, in Figure 1c, the maximum Re[ση0] appears in the zone $0<\Gamma <0.1\omega_{0}$ according to the approximate formula above and especially when $\Gamma \ll \omega_{0}$, since the damping frequency is usually much weaker than the resonance one. In this way, we can demonstrate that the lossy part of σ in (1) can take substantial values; however, this does not automatically mean significant thermal effects since the absorption is also largely dependent on the developed local fields and the angle of incidence.

### 2.2. Developed Waves

Returning to the problem in Figure 1a, let us consider two linear polarizations: one with an electric field parallel to the *y* axis (TM) and the other with a magnetic field parallel to the *y* axis (TE). The reflection coefficients $R_{\mathrm{TM}/\mathrm{TE}}$ concern the reflective waves traveling into a vacuum, while the transmission coefficients $T_{\mathrm{TM}/\mathrm{TE}}$ are the relative complex amplitudes of the transmissive wave existing into the rear half-space. After imposing the necessary boundary conditions, the respective quantities for TM fields read

(2a) $$\begin{array}{ccc} R_{\mathrm{TM}}\left(\omega \right) & = & \frac{\cos\theta -\sigma \left(\omega \right)\eta_{0}-n\left(\theta \right)}{\cos\theta +\sigma \left(\omega \right)\eta_{0}+n\left(\theta \right)}, \end{array}$$

(2b) $$\begin{array}{ccc} T_{\mathrm{TM}}\left(\omega \right) & = & \frac{2\cos\theta }{\cos\theta +\sigma \left(\omega \right)\eta_{0}+n\left(\theta \right)}, \end{array}$$

where $n\left(\theta \right)=\sqrt{\varepsilon -\sin^{2}\theta }$. The corresponding wave coefficients for the TE fields are written as

(3a) $$\begin{array}{ccc} R_{\mathrm{TE}}\left(\omega \right) & = & 1-\frac{2n(\theta )}{\varepsilon \cos\theta +\left[1+\sigma \left(\omega \right)\eta_{0}\cos\theta \right]n\left(\theta \right)}, \end{array}$$

(3b) $$\begin{array}{ccc} T_{\mathrm{TE}}\left(\omega \right) & = & \frac{2\varepsilon \cos\theta }{\varepsilon \cos\theta +\left[1+\sigma \left(\omega \right)\eta_{0}\cos\theta \right]n\left(\theta \right)}. \end{array}$$

Note that the reflective wave is always propagating since it exists into the same medium of the incoming illumination (free space), while the transmissive wave propagates only if $\varepsilon >\sin^{2}\theta$. Oppositely, if $\varepsilon <\sin^{2}\theta$, the excited fields are purely evanescent, and, therefore, they do not carry net energy within one period 2π/ω of oscillations. It should be stressed that, for the derivations above, we assume an intrinsic response of the metasurface unaltered by the substrate, namely, that the properties of σ(ω) from (1) remain unchanged. Indeed, in many experimental implementations, the behavior of a monolayer is a function of the base material that is grown atop.

To demonstrate how realistic the investigated setup is, it would be interesting to summarize certain experimental efforts that conclude with the successful fabrication of similar absorptive metasurfaces. In particular, patterned surfaces consisting of dense arrays of nanoscale silicon strips, which act as antennae, have been designed to work as transparent optical devices for the manipulation of light [29]. On the other hand, the atomic-layer deposition of titanium dioxide has been employed to fabricate metasurfaces comprising highly anisotropic nanostructures and operated efficiently across the visible spectrum [30]. In addition, even homogeneous ultrathin dielectric layers deposited on metallic substrates make perfect absorbers [31], while gate-tunable metasurfaces that enable dynamic control of the reflection are constructed with use of conducting oxide layers incorporated into reflectarray antenna elements [32]. Finally, all-dielectric metasurface arrays of scattering elements that are tuned via geometry in the mid-IR range have been materialized by implementing low-cost wafer–scale sensor fabrication on a chip [33].

### 2.3. Absorption Global Sums

The transmitted wave develops into a material with relative permittivity ε, while the respective normalized electric or magnetic field is parallel to the *y* axis and reads $F_{\mathrm{tran}}\left(z,x\right)=T\left(\omega \right)e^{-ik_{0}\left(x\sin\theta +n\left(\theta \right)z\cos\theta \right)}$, where $k_{0}=\omega \sqrt{\varepsilon_{0}\mu_{0}}$. By applying Poynting theorem, one can directly find the portion of the incident power transferred by that monochromatic wave along the *z* axis as Ptran=P0ξRe[n(θ)]|T(ω)|2, where $P_{0}$ is the total power of the incident wave. The parameter ξ varies with the type of polarization: we have ξ=1 for TM waves and ξ=1/ε for TE waves. Similarly, the incident power along the *z* direction reads $P_{\mathrm{inc}}=P_{0}\cos\theta$, while the respective reflected power takes the form $P_{\mathrm{ref}}=P_{0}{\left|R\right|}^{2}\cos\theta$. We remark that we are interested only in that component of energy flow that is normal to the boundaries; indeed, the energy equilibrium is valid only along the *z* axis since, across the tangential directions, we have the same spatial oscillations $e^{-ik_{0}x\sin\theta }$ imposed by the incident illumination.

If one integrates the relative absorbed power $\left(P_{\mathrm{inc}}-P_{\mathrm{ref}}-P_{\mathrm{tran}}\right)/P_{0}$ along entire the normalized frequency axis $\omega /\omega_{0}$, one defines the key metric regarding the global absorption sum:

(4) A=cosθω0∫0+∞1−|R(ω)|2−ξRe[n(θ)]cosθ|T(ω)|2dω,

which is also unitless. Note that the subscripts TM/TE were dropped for brevity. Remarkably, with use of the identity

(5) $$\begin{array}{c} \int_{0}^{+\infty }\frac{d(\omega /\omega_{0})}{{\left(\omega /\omega_{0}-\frac{1}{\omega /\omega_{0}}\right)}^{2}+a^{2}}=\frac{\pi }{2a}, \end{array}$$

for Re[a]>0, the integrations are carried out analytically for both polarizations but only in the case of $\varepsilon >\sin^{2}\theta$. The respective closed forms (6) are given below.

(6a) $$\begin{array}{ccc} A_{\mathrm{TM}} & = & \frac{2\pi \left(\Gamma /\omega_{0}\right){\left(\omega_{p}/\omega_{0}\right)}^{2}}{1+\frac{\sqrt{\varepsilon -\sin^{2}\theta }}{\cos\theta }}\frac{\cos\theta }{{\left(\omega_{p}/\omega_{0}\right)}^{2}+\left(\Gamma /\omega_{0}\right)\left[\cos\theta +\sqrt{\varepsilon -\sin^{2}\theta }\right]}, \end{array}$$

(6b) $$\begin{array}{ccc} A_{\mathrm{TE}} & = & \frac{2\pi \left(\Gamma /\omega_{0}\right){\left(\omega_{p}/\omega_{0}\right)}^{2}}{1+\varepsilon \frac{\cos\theta }{\sqrt{\varepsilon -\sin^{2}\theta }}}\frac{\cos\theta }{{\left(\omega_{p}/\omega_{0}\right)}^{2}+\left(\Gamma /\omega_{0}\right)\left[\frac{1}{\cos\theta }+\frac{\varepsilon }{\sqrt{\varepsilon -\sin^{2}\theta }}\right]}. \end{array}$$

Integrating along the frequency axis or certain ranges is a common practice when wideband operation is investigated and evaluated; for example, the polarization selectivity of multilayered scatterers across the visible part of the spectrum has been characterized in a similar manner [34].

Once $\varepsilon <\sin^{2}\theta$, the integrands for both sorts of waves (TM/TE) behave for ω→+∞ as $1/(C_{2}\omega^{2}+C_{1}\omega +C_{0})$ with $C_{0},C_{2}>0$; such an asymptotic trend is not very convenient in terms of fast convergence of the integral (4). Thus, the numerical integrations can be accelerated by subtracting the trivial but analytically evaluated integral

(7) $$\begin{array}{c} \int_{0}^{+\infty }\frac{d\omega }{C_{2}\omega^{2}+C_{1}\omega +C_{0}}=\frac{\pi }{\sqrt{\Delta }}-\frac{2}{\sqrt{\Delta }}\arctan\left(\frac{C_{1}}{\sqrt{\Delta }}\right), \end{array}$$

where $\Delta =4C_{2}C_{0}-C_{1}^{2}>0$. In this way, we have a reliable evaluation of the key metric *A* in (4) for both types of waves, regardless of the nature of the base where the metasurface is grown. As indicated above, the major objective of this work is the absorbing performance of causal Lorentz metasurfaces, and, thus, in our basic metric, (4) subtracts both the reflective and the transmissive power. An intriguing sequel to this study would be to investigate the global reflectivity and its balance with global transmissivity with or without thermal effects present. In this way, control over all the fundamental mechanisms of the electromagnetic interactions (reflection, transmission, absorption) via the permittivity ε or other textural and structural parameters of the problem, could be demonstrated. Therefore, we confine our analysis to examination of ohmic phenomena connected with energy-oriented, absorption-intensive applications, being separated from the research direction of analog signal processing, which involves strong reflection/transmission manipulation.

## 3. Numerical Results

### 3.1. Dielectric Substrates

**Figure 2:**
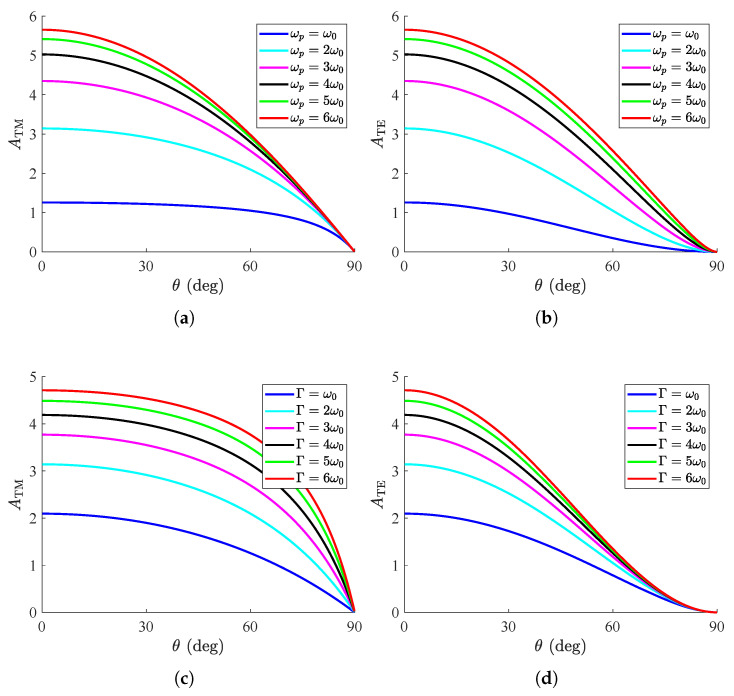
Global absorption $A_{\mathrm{TM}/\mathrm{TE}}$ as function of incident angle θ. (**a**) $A_{\mathrm{TM}}=A_{\mathrm{TM}}\left(\theta \right)$ for several plasma frequencies $\omega_{p}/\omega_{0}$ with $\Gamma =2\omega_{0}$, (**b**) $A_{\mathrm{TE}}=A_{\mathrm{TE}}\left(\theta \right)$ for several plasma frequencies $\omega_{p}/\omega_{0}$ with $\Gamma =2\omega_{0}$, (**c**) $A_{\mathrm{TM}}=A_{\mathrm{TM}}\left(\theta \right)$ for several losses frequencies $\Gamma /\omega_{0}$ with $\omega_{p}=2\omega_{0}$, (**d**) $A_{\mathrm{TE}}=A_{\mathrm{TE}}\left(\theta \right)$ for several losses frequencies $\Gamma /\omega_{0}$ with $\omega_{p}=2\omega_{0}$. Free-standing metasurface (ε=1).

In Figure 2, we depict the metric $A_{\mathrm{TM}/\mathrm{TE}}$ for free-standing structures (ε=1) as a function of the incidence angle θ for various parameters of the Lorentzian conductivity model. In Figure 2a, we observe the variation in the curves $A_{\mathrm{TM}}=A_{\mathrm{TM}}\left(\theta \right)$ for several plasma frequencies $\omega_{p}/\omega$; apparently, the recorded behavior is downward-sloping, and the absorption vanishes at the grazing angle ($\theta =90^{\circ }$). In addition, the higher the number of charge carriers ($\omega_{p}$) gets, the larger the global absorption is, while the drop is abrupt once the direction approaches the limit $\theta =90^{\circ }$. In Figure 2b, we repeat the same calculations but for TE waves, and similar conclusions are drawn; indeed, for θ=0 the values are identical to those in Figure 2a since the two polarizations become identical. The represented quantity $A_{\mathrm{TE}}$ increases slowly with $\omega_{p}$ and vanishes smoothly when the incoming ray direction becomes more oblique. This trend is justified by the limits of (6) for $\omega_{p}/\omega_{0}\to +\infty$:

(8a) $$\begin{array}{ccc} \lim_{\omega_{p}\to +\infty }A_{\mathrm{TM}} & = & \frac{2\pi (\Gamma /\omega_{0})\cos\theta }{1+\frac{\sqrt{\varepsilon -\sin^{2}\theta }}{\cos\theta }}, \end{array}$$

(8b) $$\begin{array}{ccc} \lim_{\omega_{p}\to +\infty }A_{\mathrm{TE}} & = & \frac{2\pi (\Gamma /\omega_{0})\cos\theta }{1+\varepsilon \frac{\cos\theta }{\sqrt{\varepsilon -\sin^{2}\theta }}}. \end{array}$$

In Figure 2c, the responses $A_{\mathrm{TM}}=A_{\mathrm{TM}}\left(\theta \right)$ are shown for several loss frequencies $\Gamma /\omega_{0}$, where the decreasing trend is even more abrupt compared to that in Figure 2a. Importantly, we notice that the global absorption increases for larger metasurface losses, as happens with the plasma frequency. Furthermore, in Figure 2d, TE waves are considered and the quantity declines more smoothly than in Figure 2b, while it is clear that, for increasing $\Gamma /\omega_{0}$, the effect saturates, and $A_{\mathrm{TE}}$ cannot be further enhanced. Similar to (8), the respective saturated quantities are obtained as the following perfect squares

(9a) $$\begin{array}{ccc} \lim_{\Gamma \to +\infty }A_{\mathrm{TM}} & = & 2\pi {\left[\frac{\left(\omega_{p}/\omega_{0}\right)}{1+\frac{\sqrt{\varepsilon -\sin^{2}\theta }}{\cos\theta }}\right]}^{2}, \end{array}$$

(9b) $$\begin{array}{ccc} \lim_{\Gamma \to +\infty }A_{\mathrm{TE}} & = & 2\pi {\left[\frac{(\omega_{p}/\omega_{0})\cos\theta }{1+\varepsilon \frac{\cos\theta }{\sqrt{\varepsilon -\sin^{2}\theta }}}\right]}^{2}. \end{array}$$

**Figure 3:**
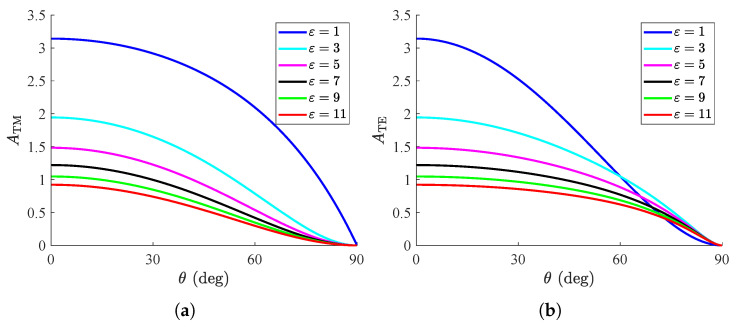
Global absorption $A_{\mathrm{TM}/\mathrm{TE}}$ as function of incident angle θ for several substrate permittivities ε. (**a**) TM polarization, (**b**) TE polarization. Metasurface parameters: $\omega_{p}=2\omega_{0}$, $\Gamma =2\omega_{0}$.

In Figure 3, the global absorption is shown with respect to the incidence angle θ for various relative permittivities ε of the substrate. In the scenario of TM polarization (Figure 3a), one observes that the response becomes more substantial for decreasing ε; additionally, contrary to what happens in other cases, the convexity of the curves does not flip in sign for ε=1. Interestingly, the opposite behavior is recorded for TE waves, namely, in Figure 3b; indeed, the second derivative $A_{\mathrm{TM}}^{\prime \prime }\left(\theta \right)$ changes sign for ε=1, which makes the setups with ε>1 better than the free-standing one (under suitably oblique incidence angles). Finally, a common characteristic of all the curves in Figure 3 is that the absorption vanishes for higher and higher ε since *A* becomes weaker and tends to zero for ε→+∞ (see, again, (6)).

**Figure 4:**
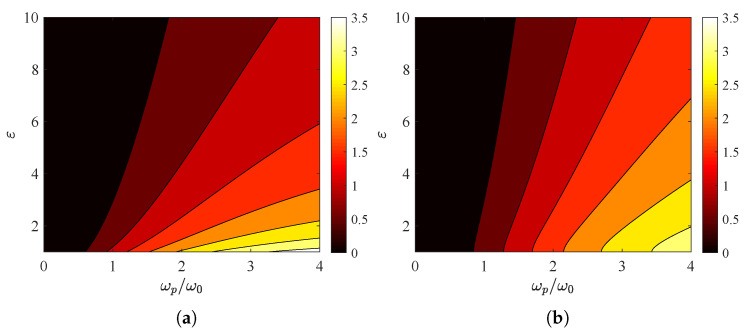
Global absorption $A_{\mathrm{TM}/\mathrm{TE}}$ in contour plots with plasma frequency $\omega_{p}/\omega_{0}$ on the horizontal axis and substrate permittivity ε one the vertical one. (**a**) TM polarization, (**b**) TE polarization. Plot parameters: $\Gamma =2\omega_{0}$, $\theta =45^{\circ }$.

In Figure 4a, we present the quantity $A_{\mathrm{TM}}$ from (6a) across a map whose horizontal axis indicates the plasma frequency of the surface conductivity and whose vertical axis shows the relative permittivity ε>1. As shown in Figure 2a,c and Figure 3a, the global absorption increases with $\omega_{p}$ and decreases with ε. The integral $A_{\mathrm{TE}}$, depicted in Figure 4b, behaves in a similar manner; nonetheless, the boost in global absorption is more substantial with respect to $\omega_{p}$, and the drop is more abrupt with respect to ε.

**Figure 5:**
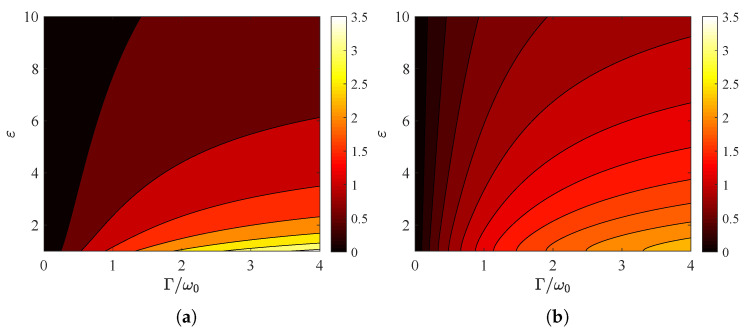
Global absorption $A_{\mathrm{TM}/\mathrm{TE}}$ in contour plots with the losses $\Gamma /\omega_{0}$ along the horizontal axis and the substrate permittivity ε along the vertical one. (**a**) TM polarization, (**b**) TE polarization. Plot parameters: $\omega_{p}=2\omega_{0}$, $\theta =45^{\circ }$.

In Figure 5a, we present the metric $A_{\mathrm{TM}}$ across a plane like that in Figure 4a but with a horizontal axis indicating the damping strength $\Gamma /\omega_{0}$. The variation is almost identical to that in Figure 4a, which means that, in this class of examples, the two frequencies ($\omega_{p}$ and Γ) have complementary roles to each other. In Figure 5b, we show the response $A_{\mathrm{TE}}$ across the same $\left(\omega_{p}/\omega_{0},\varepsilon \right)$ map; it is clear that the scores are much lower compared to the TM polarization. In addition, the negative influence of the substrate permittivity on the overall absorption is milder than in the other cases and vanishes only at a small parametric strip in the vicinity of the lossless axis Γ=0.

### 3.2. Plasmonic Substrates

**Figure 6:**
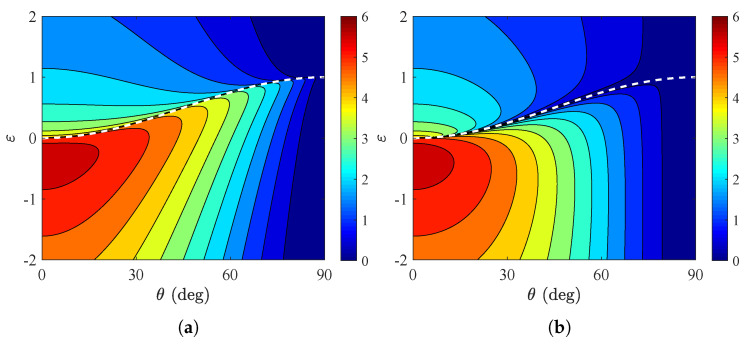
Global absorption $A_{\mathrm{TM}/\mathrm{TE}}$ in a contour plot with the incidence angle θ along the horizontal axis and the substrate permittivity ε along the vertical one. (**a**) TM polarization, (**b**) TE polarization. The white dashed line corresponds to $\varepsilon =\sin^{2}\theta$, above which only propagating waves develop. Metasurface parameters: $\omega_{p}=2\omega_{0}$, $\Gamma =\omega_{0}$.

Up to this point, we investigated scenarios with dielectric (ε≥1) bases onto which the metasurface is deposited; for this reason, the closed forms (6) were utilized. It would be, however, meaningful to also consider plasmonic lossless (ε<1) substrates and compare the absorption metrics with those of their penetrable counterparts; the evaluation of *A* is performed numerically with help from the analytical result (7). In Figure 6a, we show the global absorption $A_{\mathrm{TM}}$ across a map, with the incoming angle indicated along the horizontal axis, as in Figure 2 and Figure 3, and the relative permittivity along the vertical axis, as in Figure 4 and Figure 5. The dashed line corresponds to the equality $\varepsilon =\sin^{2}\theta$, and it becomes clear that the total absorbance is enhanced when ε<1 and especially across the zone −1<ε<0. For θ, it declines regardless of the nature of the base; in addition, the represented quantity varies mildly when one crosses the dashed line since the electric component of the incident beam is always parallel to the metasurface, no matter how oblique the illumination is.

In Figure 6b, we repeat the calculations in Figure 6a but for TE waves; once again, we realize that the peak performance is achieved for −1<ε<0 and for normal incidence (θ=0), as imposed by the factor of cosθ in the definition (4). Nonetheless, the behavior of the device changes dramatically in the vicinity of the white dashed line, where the propagating waves into the semi-infinite base become purely evanescent. Indeed, the overall absorption is locally minimized along the locus $\varepsilon =\sin^{2}\theta$ that separates the two regions, and, in the vicinity of which, a totally different behavior is recorded.

**Figure 7:**
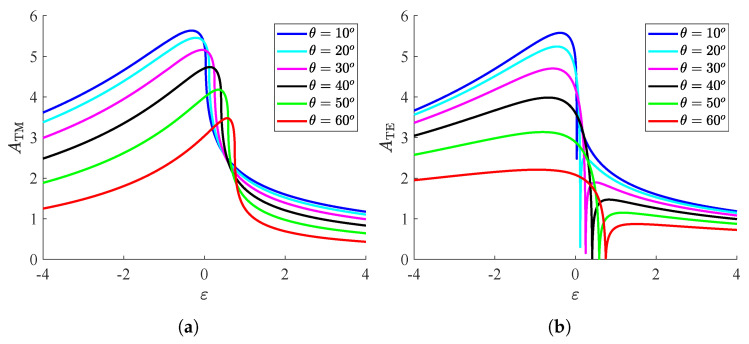
Global absorption $A_{\mathrm{TM}/\mathrm{TE}}$ as function of substrate relative permittivity ε for various incoming angles θ. (**a**) $A_{\mathrm{TM}}=A_{\mathrm{TM}}\left(\varepsilon \right)$, (**b**) $A_{\mathrm{TE}}=A_{\mathrm{TE}}\left(\varepsilon \right)$. Metasurface parameters: $\omega_{p}=2\omega_{0}$, $\Gamma =\omega_{0}$.

In Figure 7a, we sketch the curves $A_{\mathrm{TM}}=A_{\mathrm{TM}}\left(\varepsilon \right)$ for several incoming angles θ. One clearly observes the superiority of the plasmonic-based metasurfaces against the ones grown on dielectric substrates, while the maximum score occurs at higher permittivities as the incident beam becomes more oblique. It is also noted that, for increasing ε, the global absorption drops abruptly when crossing the limit $\varepsilon =\sin^{2}\theta$; such a feature demonstrates once again the absorbing preference of the setup to plasmonic bases. Importantly, when operated close to the regime of total internal reflection with $\varepsilon =\sin^{2}\theta$, the structure is particularly beneficial for switching utilities with respect to the incident direction θ.

In Figure 7b, we present the responses $A_{\mathrm{TE}}=A_{\mathrm{TE}}\left(\varepsilon \right)$ for various θ and realize that, on average, the absorbing performance is worse compared to the other type of waves (TM, Figure 7a). In particular, the larger the angle θ, the less sharp and the smaller the peak of the curve. However, the behavior of the system in the vicinity of $\varepsilon =\sin^{2}\theta$ is remarkable since the global absorption and, inevitably, the absorbed power at every single frequency almost vanish. Such an ultra-sharp response provides again a fertile ground for building extremely selective sensors that detect a specific incoming ray.

**Figure 8:**
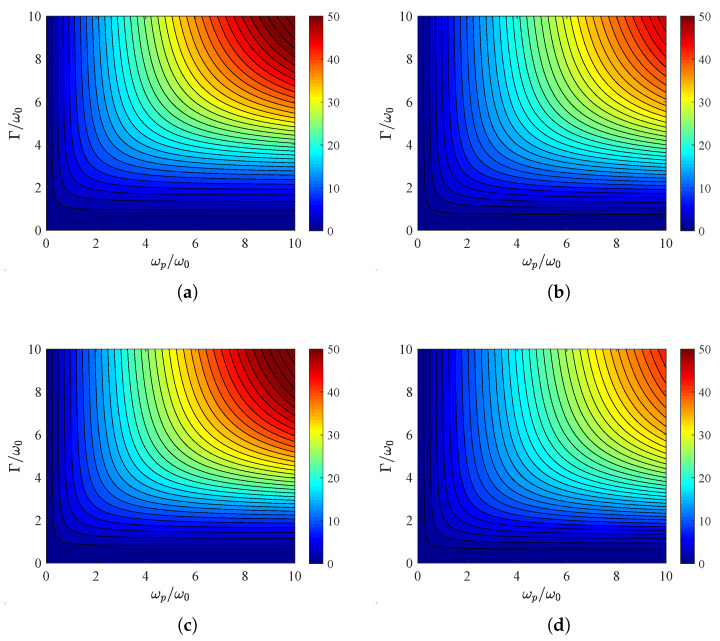
Global absorption $A_{\mathrm{TM}/\mathrm{TE}}$ in contour plots with plasma frequency $\omega_{p}/\omega$ along horizontal axis and losses $\Gamma /\omega_{0}$ along vertical one. (**a**) ε=−0.75, TM polarization, (**b**) ε=−0.75, TE polarization, (**c**) ε=−0.25, TM polarization, (**d**) ε=−0.25, TE polarization. Common incidence angle: $\theta =45^{\circ }$.

In all the previous examples, the global absorption *A* does not surpass six for neither polarization type. It is, thus, important to see what magnitudes can reach when the permittivity ε is selected within the interval −1<ε<0. In Figure 8a, we choose ε=−0.75, fix the angle at $\theta =45^{\circ }$ and present $A_{\mathrm{TM}}$ across a map $\left(\omega_{p}/\omega_{0},\Gamma /\omega_{0}\right)$ concerning the parameters of the lossy metasurface. It is stressed that the global absorption can be higher than 50 if both the normalized frequencies are substantial. In Figure 8b, where TE fields are considered, the metric is smaller than in the other type of wave in Figure 8a, as happens in most cases. In addition, the plasma frequency and the losses influence positively the absorption, as has already been reported; further enhancement in global absorption is feasible as long as a close-to-normal incidence excitation is assumed. Similar conclusions can be reached for Figure 8c,d, where the permittivity is closer to zero: ε=−0.25. The results for TM waves (Figure 8c) are even better than that in Figure 8a; nonetheless, the absorbance for TE waves is lower that that in Figure 8b. Therefore, to find the maximal value, one should optimize the response for each given metasurface with respect to the permittivity ε.

In the considered cases, we computed the global absorption sums (4) of the homogenized metasurfaces. However, the physical configuration of these setups comprises periodically placed identical nanoparticles; therefore, the respective absorptive sums $A_{\mathrm{TM}/\mathrm{TE}}$ incorporate multipolar terms, each of which corresponds to different spherical harmonics, forming Floquet–Bloch waves of different orders. In this framework, one can regulate the contribution of each term and design metasurfaces made of silicon parallelepipeds; they allow the excitation of the magnetic dipole moment and lead to the broadening and enhancement of the absorption [35]. Similarly, high-refractive-index nanostructures support optically induced electric dipole and magnetic dipole modes that can be used to control scattering and achieve narrowband absorption [36]. The rigorous mathematical treatment for nanoparticle interactions with the use of a partial wave formulation that takes into account several multipolar scattering terms has been implemented in the case of gratings from corrugated nanorods [37] and core–shell cylinders [38].

## 4. Concluding Remark

The absorption by a causal homogenized metasurface that follows the Lorentz conductivity model is summed across all the oscillating frequencies from zero to infinity. Once the metasurface is backed by a lossless dielectric substrate or is free-standing, the aforementioned integral is analytically evaluated; otherwise, a rapidly converging numerical integration scheme is followed. The defined global absorption enhances once the plasma frequency and the damping factor simultaneously increase. In a given metasurface, the highest absorbance score occurs for lossless plasmonic bases and, naturally, is a decreasing function of the incidence angle. Interestingly, it is found that absorption at all frequencies vanishes under the regime where the propagating waves turn into evanescent waves at the substrate but only for one of the two polarizations.

The same technique can be trivially expanded to cover more complicated dispersion relations for the causal metasurface, comprising sums of Lorentzians. However, a more interesting expansion of the present work would be to consider dispersive backing volumes so that the global absorption is redistributed across various bands to permit the engineering of the thermal effects. In addition, the substrate can also be considered lossy so that it contributes to the absorption of the incoming waves as well. Importantly, one may regard coupled causal metasurfaces interacting with each other to produce higher scores for absorption at the same level of total losses. The same approach can be directly utilized for producing sum rules about reflectivity and transmissivity by involving slabs instead of metasurfaces; in this way, similar algebraic steps can lead to causality implications for several operations characterizing a whole class of planar structures.

## Data Availability

The raw data supporting the conclusions of this article will be made available by the corresponding and sole author on request.
